# Highlighting plant science with a GENETICS and G3 series on Plant Genetics and Genomics

**DOI:** 10.1093/genetics/iyad003

**Published:** 2023-02-09

**Authors:** Thomas E Juenger, Andrea L Sweigart, Jianming Yu, James Birchler

**Affiliations:** Department of Integrative Biology, University of Texas at Austin, 2415 Speedway, Austin, TX 78712, USA; Department of Genetics, University of Georgia, 120 E. Green Street, Athens, GA 30602-7223, USA; Department of Agronomy, Iowa State University, 2401 Agronomy Hall, 716 Farm House Lane, Ames, IA 50011-1051, USA; Biological Sciences, University of Missouri at Columbia, 105 Tucker Hall, Columbia, MO 65211-7400, USA

Plant science has generated many discoveries and advances in genetics and genomics research. These contributions reflect the ingenuity and rigor of the plant science community as well as the rich diversity of plants and their biology. Plants are also vital in our daily lives as a source of food, fiber, and fuel and as a key component of the global carbon cycle. As such, the importance of plant research should not be underestimated, especially as society copes with changing climate and its impacts to crop productivity and sustainable energy.

GENETICS has a long history of publishing key papers in plant genetics. These include Barbara McClintock's groundbreaking research on maize genetics and cytogenetics (McClintock 1929; McClintock and Hill 1931), including the discovery of unstable mutations (McClintock 1953); Marcus Rhoades’ discovery of what we now call meiotic drive (Rhoades 1942); the discovery that UV radiation causes mutations in DNA (Stadler and Uber 1942); early work on quantitative genetics (East 1916), hybrid vigor (Jones 1917; East 1936; Crow 1948; Shull 1948), and genetic design (Hayman 1954; Jinks 1954); the first example of parental gene imprinting (Kermicle 1970); and many papers providing important insights into plant domestication and crop improvement (e.g. Doebley and Stec 1991; Stuber *et al*. 1992; Doebley *et al*. 1995; Burke *et al*. 2002; Simons *et al*. 2006). GENETICS has also fostered the development of *Arabidopsis* as a model plant with the publication of the first *Arabidopsis* mutant screen (Rédei 1962), and over the years, the characterization of many key mutants and molecular pathways. Genetics Society of America (GSA) journals continue to publish many advances in plant science, including genomic studies of hybrids and polyploids, studies of plant evolution and quantitative genetics, and modern advances in plant breeding.

To showcase Plant Genetics and Genomics, the GSA has launched a special collection of publications in the GSA Journals GENETICS and G3: *Genes|Genomes|Genetics*. This editorial accompanies 14 papers highlighting exciting advances being made in a range of Plant Science fields.

Like many areas of biology, new genomic tools and DNA sequencing advances have had a dramatic impact on plant science. In particular, next-generation sequencing technologies, especially long-read sequencing and new genome assembly approaches, are changing the scope of questions that researchers can address. Rapid assembly and annotation of complex genomes, the generation of intraspecific pangenomes, and the commonplace screening of genome-wide SNP polymorphisms are developments that are advancing our understanding of genetic diversity within model systems, crop plants, and native species. The influence of these advances was apparent in a number of manuscripts in the Series.

Four manuscripts from the Series focus on the mechanisms, evolution, and impacts of polyploidy and chromosomal-scale variation in plants. Scarlet *et al*. (2023) studied the origin and evolution of the natural allotetraploid grass *Brachypodium hybridum* using PacBio sequencing. They compared 2 independently derived *B. hybridum* accessions to their deeply diverged diploid progenitor species *Brachypodium stacei* and *Brachypodium distachyon* to test the “genome shock” hypothesis: the idea that whole-genome duplications may induce chaotic genome restructuring. Their data provide little support for the “genomic shock” hypothesis and instead suggest a more nuanced model of relaxed selection and transposable element effects on gene loss and subgenome dominance in *Brachypodium*. Oruganti *et al*. (2023) explore the role of hybridization and polyploidy in gene flow, introgression, and reproductive isolation in the genus *Arabidopsis*. They experimentally test whether autoallohexaploids can serve as an intermediate bridge between diploids and polyploids. PacBio sequencing and optical mapping allowed the assembly of *Arabidopsis suecica* allopolyploid and permitted the use of short-read sequencing of hexaploidy progeny—the multigeneration product of *Arabidopsis thaliana* (2N) × *A. suecica* (4N) hybridization—to detect meiotic recombination, aneuploidies, and homologous exchanges between the progenitor subgenomes. Their results show that interploidy crosses can form viable and recombinant progeny and, therefore, may serve as a bridge between diploid and polyploid species. Sharma and Peterson (2023) investigate the role of transposable element insertion in maize as a driver of complex chromosomal rearrangements that have an impact on *cis*-regulatory variation. They use a creative *p*1/*p*2 gene enhancer system to detect structural rearrangements that were confirmed using Chromosome Conformation Capture (3C) experiments. Their results show that progressive rearrangements can form complex alleles resulting in diverged expression patterns. Guo *et al*. (2023) also study complex chromosomal rearrangements using a MEIOTIC ASYNAPTIC MUTANT (*asay1*) background of *A. thaliana*. They cross *asay1* with a Columbia wild-type line and recover a progeny exhibiting chromoanagenesis, a phenomenon characterized by localized chromosomal shattering and reorganization. Overall, they observed hundreds of novel DNA junctions falling within the shattered region of Chromosome 1, with the pattern of junctions suggesting random reassembly of the pieces and a completely reorganized chromosomal region. Their study provides a first step in deciphering the molecular mechanisms leading to chromoanagenesis and its potential role in plant genome evolution. Together, these papers provide new insights into the mechanisms and role of ploidy and chromosome-scale variation in plants and give a glimpse of a rapidly expanding field.

A second theme of the series arises from studies of the genetic basis of quantitative traits. Here, 7 manuscripts use genome-wide molecular markers to describe diversity and map genomic regions contributing to trait variation. Carlson *et al*. (2023) present a comprehensive quantitative genetic analysis of agronomically important traits in cultivated oat (*Avena sativa*) using an impressive array of 9 multiparent families and a TILLING population. They identify a number of marker-trait association hotspots, likely the result of pleiotropic gene action, and discover a notable link between panicle architecture and disease susceptibility. Haugrudet *et al*. (2023) study the genetics of yield-related traits in an emmer × durum wheat population grown in field and greenhouse conditions. They characterize the unique and shared genetic architecture across traits and environments and identify a number of robust and environmentally stable alleles contributed from the emmer wheat parent. Similarly, Rahimi *et al*. (2023) use genome-wide association studies (GWAS) and a meta-analysis of existing QTL data to study early vigor and grain yield QTL under simulated water deficit in bread wheat. These 3 studies provide a foundation for oat and wheat improvement and expand our understanding of the genetic architecture of key agronomic traits in 2 important cereal crops. Okunlola *et al.* (2023) use GWAS to identify genomic regions that have an impact on resistance to *Striga hermonthica*, an endemic parasitic weed of maize in West Central Africa. Here, the researchers expose maize lines to standardized *Striga* exposure in the field and evaluate the number of emerged *Striga* and host plant damage data at 2 sites over multiple years. The authors find a relationship between the number of emerged *Striga*, host plant damage, and grain yield, and genome-wide association scans identify a number of SNPs underlying *Strig*a resistance traits. These findings may afford opportunities for molecular breeding of *Strig*a resistance in maize. Palmer *et al*. (2023) study the genetic basis of hybrid vigor and rootstock development in pistachio orchard trees. The challenging heterozygous and outbred nature of the material is addressed by generating chromosome-scale assemblies of the *Pistacia atlantica* (female) and *Pistacia integerrima* (male) parental trees of an F_1_ mapping population. Genetic mapping analyses identify loci associated with tree size and shape, sex, and precocity as well as implicate a strong candidate for the ZZ/ZW sex chromosomes. Woods *et al*. (2023) use genome resequencing to elucidate the recent evolutionary and domestication history of *Cannabis sativa*, with a focus on disentangling evolutionary forces that have influenced the divergence of domesticated and feral (escaped domesticated lineages) populations. Population structure and evolutionary analyses support an Asian origin of *C. sativa* and provide some insight into the origin of marijuana- and hemp-type accessions. Selection analyses based on polymorphism data implicate the action of positive and balancing selection on a number of genomic regions. These regions may have been targets of domestication selection and signals of local adaptation in the US feral populations. Brůna *et al.* (2022) present a Genome Report detailing a high-quality chromosome-scale assembly and annotation of the diploid blackberry germ-plasm accession “Hillquist” (*Rubus argutus*). The utility of the resource is demonstrated by its support of the first genotyping-by-sequencing-based linkage map of tetraploid blackberry and the identification of possible candidate genes for annual fruiting. This set of papers provides a clear picture of the importance and rapid adoption of marker-based methods and the importance of these tools in applied plant science.

Three papers use molecular manipulations to evaluate the effect of candidate genes on plant phenotypes. Andleeb *et al*. (2023) investigate the role of an NAC transcription factor (NAM-B1) in remobilization of nitrogen and micronutrients from vegetative tissues to grain in wheat (*Triticum aestivum*). They conduct a time-series expression analysis of nitrogen remobilization in RNAi lines that have reduced NAM gene expression. They discover that nitrogen metabolism genes exhibit earlier and stronger expression in wild-type compared with mutant lines, providing insight into NAM-regulated and NAM-independent candidate genes and pathways involved in senescence and remobilization. Lemus *et al*. (2023) investigate genes involved in buffering environmental and genetic variation in the model plant *A. thaliana*. Their project centers on the potential role of Argonaute 1 (AGO1) in phenotypic buffering through its role in microRNA-mediated pathways controlling gene expression. They show that *AGO1* buffers environmental and genetic variation for the relationship between rosette leaves and flowering time and use a creative bulk segregant analysis to identify *HUA*2 as an AGO-1-dependent buffering locus. Finally, Ando *et al*. (2023) study the role of *ETHYLENE INSENSITIVE* (*EIN2*) in seed development and size in *Arabidopsis*. They find that *EIN2* is maternally expressed in endosperm through seed development, that methylation plays a role in the pattern of maternal-specific expression, and that *EIN2* affects the temporal regulation of endosperm cellularization with consequences for seed size. This set of papers highlights functional genetic work in model and crop species, which helps to elucidate molecular mechanisms linking genotype and phenotype—a research program that we anticipate will continue to increase with improved transformation methods and genome assemblies.

We are pleased with the community response to our call for papers and hope that this outstanding set of publications showcases progress as well as motivates additional submissions. This is an open call for papers, and we encourage authors to continue to submit their work to GENETICS and G3. We would especially welcome commentaries and perspectives about rapidly advancing fields in Plant Science. New submissions can be directly submitted to either of the two journals; manuscripts will be subject to standard peer review and editing, and accepted articles will be added to the online collection.
